# Data-driven networking of global transcriptomics and male sexual development in the main malaria vector, *Anopheles funestus*

**DOI:** 10.1038/s41598-023-43914-0

**Published:** 2023-10-05

**Authors:** L. L. Koekemoer, M. Hajkazemian, J. W. Zawada, M. Mirzaie, Y. L. Dahan-Moss, S. N. Emami

**Affiliations:** 1 Wits Research Institute for Malaria, School of Pathology, Faculty of Health Sciences, University of the Witwatersrand, Johannesburg, South Africa; 2 Centre for Emerging Zoonotic and Parasitic Diseases, National Institute for Communicable Diseases, a Division of the National Health Laboratory Service, Johannesburg, South Africa; 3 Department of Molecular Biosciences, Wenner-Gren Institute, Stockholm University, Stockholm, Sweden; 4 Department of Pharmacology, Faculty of Medicine, University of Helsinki, Helsinki, Finland; 5 Molecular Attraction AB, Elektravägen 10, Hägersten, 126 30 Stockholm, Sweden; 6 Natural Resources Institute, FES, University of Greenwich, London, UK

**Keywords:** Reproductive biology, Transcriptomics

## Abstract

Deaths from malaria remain staggering despite global support that drives research into new territories. One major gap is our understanding of the sexual biological aspects of the male mosquito, which maintain the vector population solidity. Although *Anopheles funestus s.s.* is an extremely efficient African vector, little is known about the network between its sexual physiology and gene expression. The Culicidae male’s sexual maturity involves a suite of physiological changes, such as genitalia rotation that is necessary for successful mating to occur. We show that mating success is guided by genes and physiological plasticity. Transcriptome analysis between newly emerged males (immature) versus males with rotating genitalia (maturing) provides insight into possible molecular mechanisms regulating male sexual behaviour. Putative transcripts that were associated with male sexual maturation were identified and validated. The discovery of the functions of these transcripts could lead to identifying potential targets for innovative vector control interventions, and mosquito population suppression.

## Introduction

Approximately 241 million cases of malaria occurred worldwide in 2020 resulting in an estimated 627,000 deaths, with 94% of these occurring in the WHO African region. In 2020, publication of a modelling analysis that quantified the likely impact of the COVID-19 pandemic predicted that malaria mortality in sub-Saharan Africa would increase, relative to a 2018 baseline^1^. One of the major malaria vectors in Africa belongs to the *Anopheles funestus* group^2^. *Anopheles funestus s.s.* is an efficient vector of malaria parasites due to its anthropophilic and endophilic behaviour combined with its wide distribution across tropical and subtropical Africa^2,3^. *Anopheles funestus* has been thoroughly studied regarding its insecticide resistance profile and associated molecular resistance mechanisms^3–5^. There have also been several studies to understand the biology and behaviour of *An. funestus*^6–12^. However, the information on molecular mechanisms by which the sexual physiological traits of the male mosquito are modulated has received limited attention. This is likely because it is more effective to control mosquito populations by limiting the reproductive success of females rather than males.

Male Culicidae undergo various physical changes during the day after pupal enclosure and before they are sexually mature and able to mate with females^13^. These changes include, amongst others, the rotation of the claspers (also referred to as genitalia rotation), the ability of antennal fibrillae to erect, as well as complete maturation of the male internal sexual organs (accessory glands and gonads)^13–17^. The male genitalia of adult mosquito are located in the abdominal segments located on segment 8–10. These segments include the claspers that are tipped with claws^14,18,19^. The claspers must rotate by 135–180° to enable the male to grasp the female during copulation^8,14,17^. During mating, the female and male grasp each other in an ‘end to end’ position during flight, and the male will release the female after mating^19^. Clasper (genitalia) rotation has been classified into five stages (S0–S4), depending on the position of the claws^8^. Stage 0 (S0) is unrotated genitalia, S1 rotation is between 1 and 45°; S2–S4 are identified by rotation of 45–90°, 90–135° and 135–180° respectively. Full rotation can take between 14 and 36 h depending on the environmental temperature^8^. *Anopheles funestus* male swarms are predominately of sexually mature males with complete genital rotation (S4)^10^.

Historically, the importance of muscle tissue is considered to be the only important feature of mosquito genitalia rotation^20^. Knowledge of insect muscle tissue has expanded over the last few decades, and apart from its role in physiological processes like genitalia rotation, it is a large store and consumer of energy^13,21^. The inversion of the insect terminal abdominal segments or genitalia rotation is caused by the contraction of the intersegmental muscles. These muscles are known as rotational muscles and have unique properties as super-contracting muscles (energy efficient)^13^.There are two pairs of opposed rotational muscles in the adult male mosquito. One pair is situated dorsally and the other is situated ventrally^13^. During the rotation event, one member of each rotation pair shortens while the other is extended. The stretched muscles never contract, and the contracting muscles shorten only once^13^.

The direction of the rotation is random, either clockwise or anti-clockwise. This suggests that the rotation direction is determined by whichever muscle contracts first or the more strongly^20^, a major difference from *Drosophila* males, where the direction of rotation is critical for successful mating^22–24^. It is still unknown if the rotational direction is also relevant in determining successful mating in mosquitoes. According to Chevonne and Richards^20,25^ the arthrodial membrane, a tough non-calcified flexible cuticle between the 7th and 8th segments can greatly stretch during rotation and the mesocuticle of adjacent sclerites showed realignment of fibres^26^. The arthrodial membrane comprises of proteins with a Rebers-Riddiford (RR) consensus sequence and specifically RR-1, which allows for chitin binding via this consensus sequence^26–28^. In addition to the physical changes mentioned above, the male also needs to acquire the ability to erect its antennal fibrillae^18,29–36^. After emergence, the male mosquito has the capability of erecting the fibrillae to an almost perpendicular angle relative to the flagellum. Sexually-mature males’ flight activity associated with mating (swarming) cause the antennal fibrillae to become erect during this period^29–35^. Male antennal sensory organs (chemoreceptors) allow them to respond to aggregation pheromones released by other males, which encourages them to join in swarming activity^12^. The same authors also showed that these pheromones also attract females during swarming and increase successful mating^20,25^.

The molecular and physiological modifications during adult male maturation are unknown in *An. funestus* and other species of Culicidae. However, there has been some progress in understanding the phenomena of male genitalia rotation in Diptera using *Drosophila* males as models. *Drosophila* males undergo a characteristic 360° genitalia rotation, as opposed to the 180° genitalia rotation reported in mosquitoes. Macías et al.^37^ described the role of platelet vascular factor (PVF) receptors as well as specific apoptotic genes (which encode for phosphatases) in the male genitalia rotation. In addition, myosin, part of a superfamily of actin-based motor proteins, was also found to be an essential protein during this genitalia rotation process^23^. This protein family plays a role in muscle contraction and has ATPase activity^21^.

Transcriptomics studies using male-specific tissues (accessory glands/testis), expanded knowledge of the Diptera male biology. Wei et al.^38^ used transcriptomic analysis to identify genes from male accessory glands and the ejaculatory duct in the oriental fruit fly (*Bactrocera dorsalis*). Apart from a large array of immunity genes, the authors also identified important reproductive genes, such as the juvenile hormone and CYP302A1, the ecdysteroidogenic P450, (encoded by *disembodied*), in the oriental fruit fly^38^. Spermatogenesis is central to male maturation and a few studies using reproductive-specific tissue identified key male-biased genes associated with the reproduction process. The bulk of these genes were identified in *Drosophila melanogaster* and had functional activity that included siRNA binding, electron carrier, male fertility, and motile cilium assembly^39^. It seems that there is a lack of research when it comes to the spermatogenesis and male reproductive biology of disease-carrying mosquitoes. There are only a limited number of studies on this topic. It is important to continue exploring and understanding the reproductive biology of mosquitoes to better prevent and control the spread of diseases they may carry. Taxiarchi et al.^40^ studied testis cell lines and identified key molecular stages of sperm development and maturation in *An. gambiae* during spermatogenesis^40^. Antennae-specific studies identify specific odorant-binding proteins (OBPs) in *An. gambiae*^41^ as well as a few key sex chemosensory genes from *An. coluzzii*^42^. These included specific olfactory receptors (*ORs*), ionotropic receptors (*IRs*), gustatory receptors (*GR*) and genes for odorant-binding proteins (OBPs). One *GR* (*GR33*) and three OBP*s* were male-biased specific^43^. These transcriptomic studies provided valuable insight into the biology of the male mosquitoes, but there are major gaps in our understanding. Male maturation encapsulates a landscape of changes, some of these physical i.e. the rotation of the genitalia in tissue tissue-specific manner, however, other changes might not be tissue-specific and not necessarily just associated with the last abdominal segments or the antennae. For this reason, a whole-body transcriptome study was conducted. This allowed for the identification of changes in immunity, metabolic enzymes, etc. A comprehensive molecular pathway that matches the physiological modifications during adult male maturation, , is still undescribed in *An. funestus* and other species of Culicidae. The aim of this study was to identify differentially expressed genes (DEG) or transcripts between immature (S0) and maturing males (S3) in order to gain valuable insight into the complex molecular mechanisms that might be at play as the males age in the first few hours after eclosure.

## Results

### The *An. funestus* male transcriptome assembly and analysis

**Figure 1:**
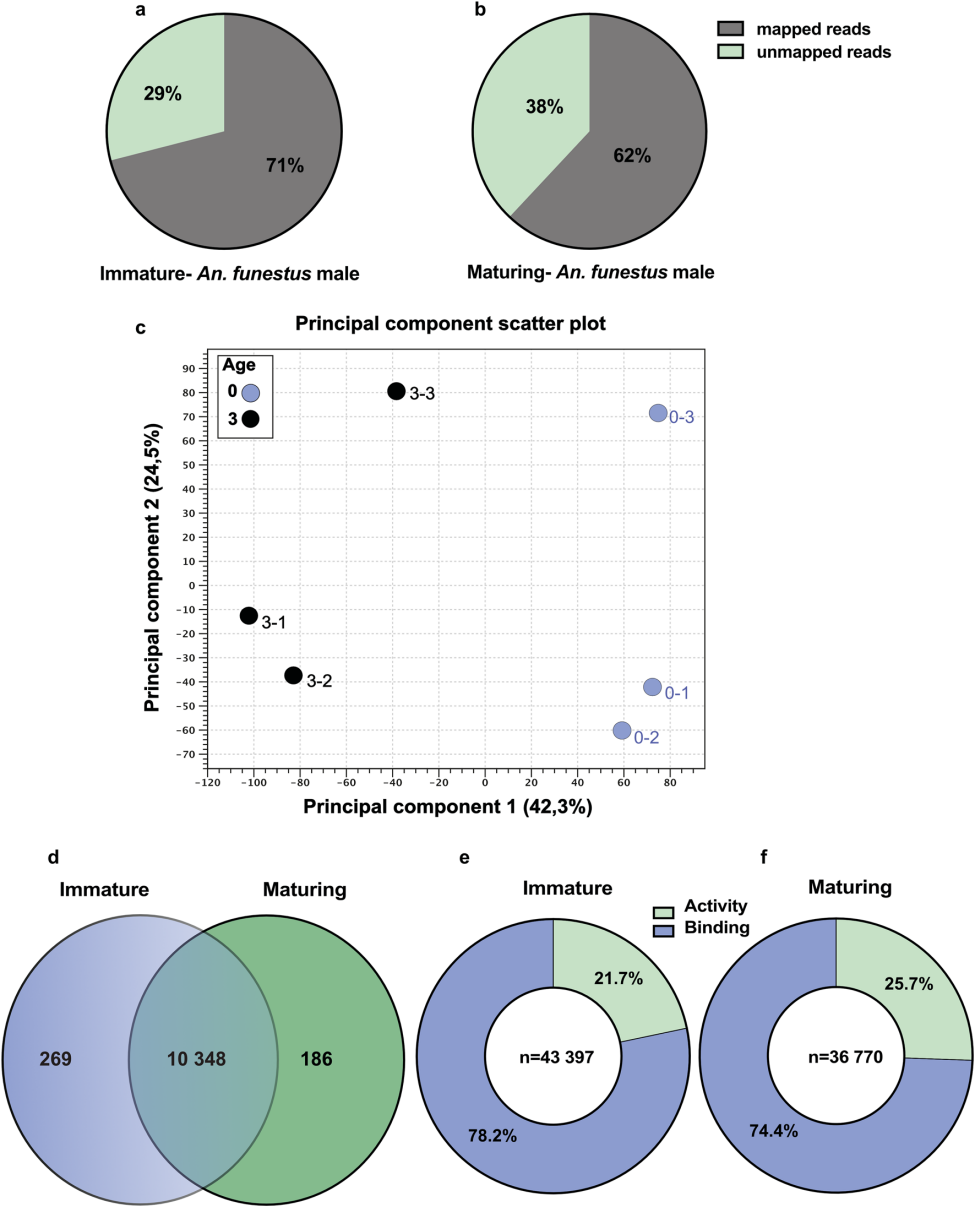
Male maturation stages affect transcript abundance in *An. funestus*. Overall transcriptome abundance. (**a**–**b**) The *An. funestus* males percentage of mapped and unmapped reads in immature and mature samples in total. (**c**) A principal component analysis representing the overall transcript abundance in the immature, (S0), and maturing (S3) *An. funestus*. The two major principal components explain that 66.8% of the variance for the three biological replicates of each maturation age and biological replication, as indicated by different colours. (**d**) sexual maturation process induces differential transcript abundances. The proportional Venn Diagram shows a pairwise comparison (n = 10,769) between the whole-body transcriptomes of immature and maturing males. Overlapping regions represent the subsets of transcripts that are shared between the different maturation stages. Significant enhancement was determined as a fold change of greater than 1.5, and an FDR-corrected *p* value < 0.05 in pairwise comparisons. (**e**–**f**) proportion of genes with differentially abundant transcripts in the whole-body of control group [immature (S0)] and maturing (S3) males were classified by a level 3 molecular functions GO. These transcripts were compared under two big classification of activity and binding-related transcripts. The legend in each pie graph indicates the GO terms representing the percentage of the total differentially abundant transcripts in these two major classified GOs for each maturation stage.

Raw data showed the average number of bases sequenced between the three biological replicates of *An. funestus* males with no genitalia rotation (referred to as immature) and males with genitalia rotated between 90 and 135° (referred to as maturing) were 10.03 and 9.71 Gb respectively. Both sample sets displayed similar GC content (44.92 and 42.72% respectively) and 95.87% of the immature *An. funestus* males and 95.92% of the maturing males had a phred quality score of ≥ 30. This is indicative that the accuracy of each nucleotide is 99.9%, which is the measure of the quality of the identification nucleobases from sequencing. After the removal of artefacts such as low-quality reads, adapter sequences, PCR duplicates and contaminant DNA, the average number of bases sequenced between the three biological replicates of *An. funestus* immature and maturing males were 9.86 and 9.57 Gb respectively. The trimmed cDNA reads obtained from the RNA sequencing were mapped to the reference genome of *An. funestus*, (AfunF3.1; FUMOZ) available on VectorBase (www.vectorbase.org)^44,45^. Overall, the immature *An. funestus* males had 71% reads mapped, while maturing *An. funestus* males had 62% mapped (Fig. 1a–b; Supplementary Table 1).

### Sexual maturity modulates global transcript abundance in male *An. funestus*

Quantitative paired-end sequencing of whole male bodies from each of the libraries generated an average mapping of 50 671 889 cleaned reads per library. Out of the total reads, 14 176 coding genes were annotated in the genome of *An. funestus* (FUMOZ). A minimum cut-off for each gene of one transcript per million (1 TPM) was introduced and all 14 176 transcripts were reliably detected above the threshold 1 TPM mapped reads, demonstrating an adequate level of coverage. A principal component analysis (PCA) of the whole-body transcripts was conducted to assess the overall variation among the transcriptome libraries (3 replicates per male group) and specifically to identify and quantify any overall differences between the two different temporal maturation groups (Fig. 1c). All of the biological independent replicates of the same maturation time clustered together in the principal component space, indicating that no significant differences were induced into the libraries by handling and processing. According to the PCA, maturation time accounted for 42.3% of the variation among the libraries (PC1), while biological replication accounted for 24.5% of the variance (PC2). This suggests that both factors play a significant role in determining the differences between the libraries. (Fig. 1c).

The degree and direction of variation in the PC1 is interesting, as it correlates with the observed immature *An. funestus* male adults and maturing adults (Fig. 1c). In total, 10 803 transcripts were identified in both the immature and maturing male adults, of which 10 348 transcripts were shared between them. Among all the transcripts that were significantly differentially regulated between immature and maturing males, a total of 186 transcripts demonstrated increased abundance in the maturing *An. funestus* male adults compare to the immature adult males through our differential expression analysis. A total of 269 transcripts were more abundant in the immature adult males compared to maturing adults (Fig. 1d). When the male mosquito matures, the predominant molecular function that was regulated at a gene level, shifted from binding towards activity (Fig. 1e–f), and in the maturing males, activity-related genes took precedence (around 4% statistically significant enhancement). Our transcriptome profiling reveals differences in transcript abundance in the reproductive organs as well as in other body tissues such as in the central nervous system (CNS) to reflect the global changes in the male adult during the two time periods evaluated here.

### Sexual maturation affected the gene expression profiles of *An. funestus* males

Comparison of the whole-body transcriptomes of immature males and maturing cohorts showed that maturation accounted for more of the variation described by the PCA on the PC1 axis (Fig. 1c). The enriched gene ontology (GO) terms were identified using PANTHER with adjusted *p* value < 0.05, as described in the method section. The significance of the statistical overrepresentation test was analysed by applying Fisher’s Exact test with False Discovery Rate correction, and the overall read quality, total bases, total reads, GC (%) and basic statistics were calculated. To address the statistical overrepresented features, we implemented measures to improve the overall read quality. We filtered out low-quality reads by utilizing Trimmomatic with the Maximum Information analytical tool. This allowed us to obtain more reliable and accurate results. Functional annotation using GO analysis classifies the predicted function of these genes (Supplementary Fig. 1a–d). Based on homology, genes were divided into three GO classes: molecular function (containing mainly ‘binding’ and ‘catalytic’ activity), cellular components and biological processes. The overall proportion of these genes in each molecular function category remained relatively consistent during the maturation process (Supplementary Fig. 1c–d). Under the molecular binding category, the most abundant class (20.37%) of transcripts in young immature males were classified under molecular function (GO:0003674; Supplementary Fig. 1a–b). This specific GO term is recommended for the annotation of gene products whose molecular function is still unknown and indicates that no information about the molecular function was available at the time of analysis. The majority of the abundant transcripts (> 76%) were functionally classified as macromolecule and compound membrane-bounded organelle (GO:0043227; Supplementary Fig. 1c–d). Enzyme regulator activity (GO:0030234) represented > 55% of transcripts in the immature males, while it was only 2.51% in the maturing males. Enzyme activity (GO:0008047), ATPase activity (GO:0016887), and lipid transporter activity (GO:0005319) classes were more abundant in maturing male compared to the younger individuals (Supplementary Fig. 1c–d).

**Figure 2:**
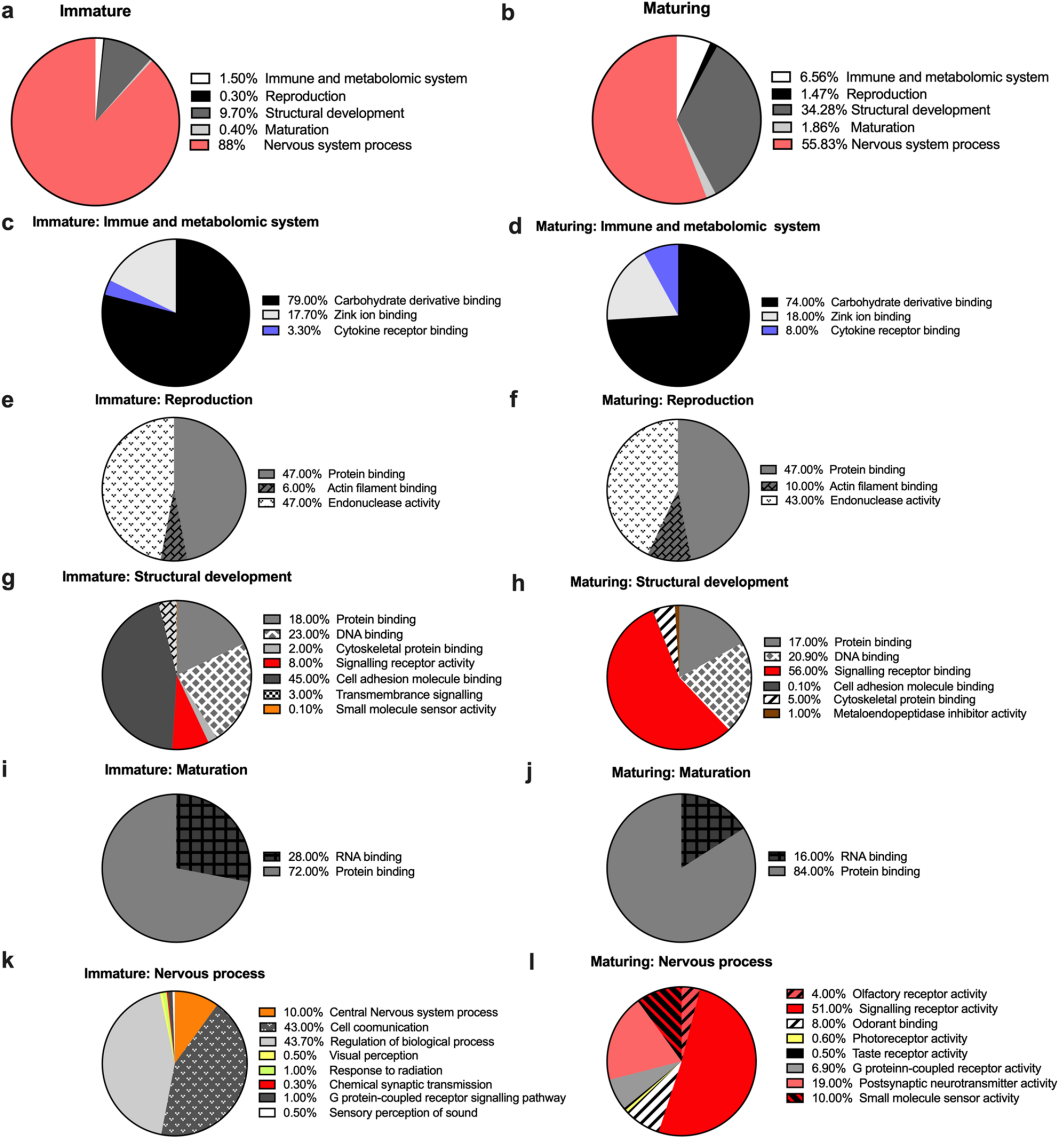
*Sexual maturation* stages in male mosquito affects the functional ontology of differentially abundant transcripts. (**a**–**l**) Proportion of genes with differentially abundant transcripts in the whole-body of immature (S0) and maturing (S3) males were classified by a level 1–2, biological processes and cellular components gene ontology classification (GO). (**a**–**b**) These transcripts were compared under five big classification of immune and metabolomic system, reproduction, structural development, maturation, and nervous system process related transcripts. (**c**–**l**) The legend in each pie graph indicates the cellular component GO terms representing the percentage of the total differentially abundant transcripts in these five major classified biological process GOs for each sexual maturation stage, S0 and S3.

The five most represented biological processes classed in the comparisons between the immature and maturing groups whole body transcriptomes, were immune and metabolomic system (GO:0002376), reproduction (GO:0000003), structural development (GO:0048856), maturation (GO:0021700), and nervous system process (GO:0050877). The significant changes in biological processes during the male mosquito maturation were structural development (GO:0048856) and the nervous system process (GO:0050877). The transcripts in these two classes differ in abundance in the whole body of mosquitoes in maturing males compared to immature males (Fig. 2a–b).

### Effect of male sexual maturity on immune and metabolic system processes

When comparing the transcriptomes of immature to maturing cohorts, carbohydrate derivative binding (GO:0097367), zinc ion binding (GO:0008270), and cytokine receptor binding (GO:0005126) accounted for more of the variation described by the GO analysis (Fig. 2c–d). As the male progresses through the genitalia rotation and maturation, sugar feeding is presumably one of the main activities; however, the proportion of differentially abundant transcripts for the cytokine receptor binding category in maturing males were 2-fold higher (3.30–8%; Fig. 2c–d). The zinc ion binding transcript abundance showed a slightly lower proportion in immature males.

### Effect of male sexual maturity on reproduction

During aging from S0 to S3, the regulation of protein binding (GO:0042802), actin filament binding (GO:0051015) and endonuclease activity (GO:0004519) genes commenced. However the genes regulating actin filament binding were shown to be differentially more abundant in maturing males. The main clusters in the molecular function classification were cytoskeleton genes and energy production genes (Fig. 2e–f).

### Effect of male sexual maturity on structural development genes

The overall trend in structural development gene abundance was denoted by a sharp increase with male sexual maturity (Fig. 2g–h), although this was generally less pronounced in protein (GO:0005515) and DNA (GO:0003677) binding. Interestingly, the number of signalling receptor activity abundant transcripts (GO:0038023) in the functional class of structural development were regulated in both immature and maturing samples, while those in the signalling receptor activity class were significantly 7-fold abundant in the maturing samples (Fig. 2g–h). The cell adhesion molecule binding (GO:0050839), small molecule sensor activity(GO:0004888), and transmembrane signalling (GO:0140299) transcripts were more abundant in the immature samples.

### Effect of male sexual maturity on the maturation process

The overall trend of maturation-related gene abundance denoted as RNA binding (GO:0003723) and protein binding (GO:0005515), showed an increase in protein binding as male adults aged through the maturation stages (Fig. 2i–j).

### Effect of male sexual maturity on the nervous system

During male genitalia rotation and sexual maturity, the regulation of nervous system process was highly maintained; however, the proportion of differentially abundant transcripts for central nervous system (GO:0050877), cell communication (GO:0007154), visual perception (GO:0007601), response to radiation (GO:0009314), chemical synaptic transmission (GO:0099565), and sensory perception of sounds (GO:0007605) was higher in the immature stage (S0). In contrast, there was a significant shift in transcript abundance of the peripheral nervous system in maturing males at stage S3, such as the olfactory receptor activity (GO:0004984), signalling receptor activity (GO:0038023), odorant binding (GO:0005549), photoreceptor activity (GO:0009881), taste receptor activity (GO:0008527), postsynaptic neurotransmitter activity (GO:0098960), and small molecule sensor activity (GO:0140299) (Fig. 2k–l).

### Regulation of sexual maturation genes in *An. funestus* males

When evaluating the abundant transcripts involved in biological processes, as expected, the majority of transcripts are categorised as developmental processes (n = 344), neural (n = 118), immune system (n = 49), reproductive (n = 31), olfactory (n = 17), sex tissue (n = 17) and other genes (n = 49) (Supplementary Fig. 2). Among the ‘developmental prediction’, both transcription and translational machinery were evident. Furthermore, a large number of DEGs genes encoding ribosomal proteins were identified, such as the ribosomal protein 27 (*RSP27*) and the large ribosomal subunit transcripts (*RPLs*), which displayed different differential expression between immature and maturing males (Supplementary Fig. 2). In addition, pyruvate kinase (PyK) enzymes involved in muscle development, glycolysis and glucose homeostasis, catabolite repression control (Crc) proteins, involved in suppressing expression of several genes, as well as signal transduction pathways were also identified to be differentially expressed between immature and maturing males^46^. The *numb* gene proteins (membrane-associated, phosphotyrosine-binding (PTB) domain-containing protein) involved in cell differentiation were also identified as DEGs^47^.

**Figure 3:**
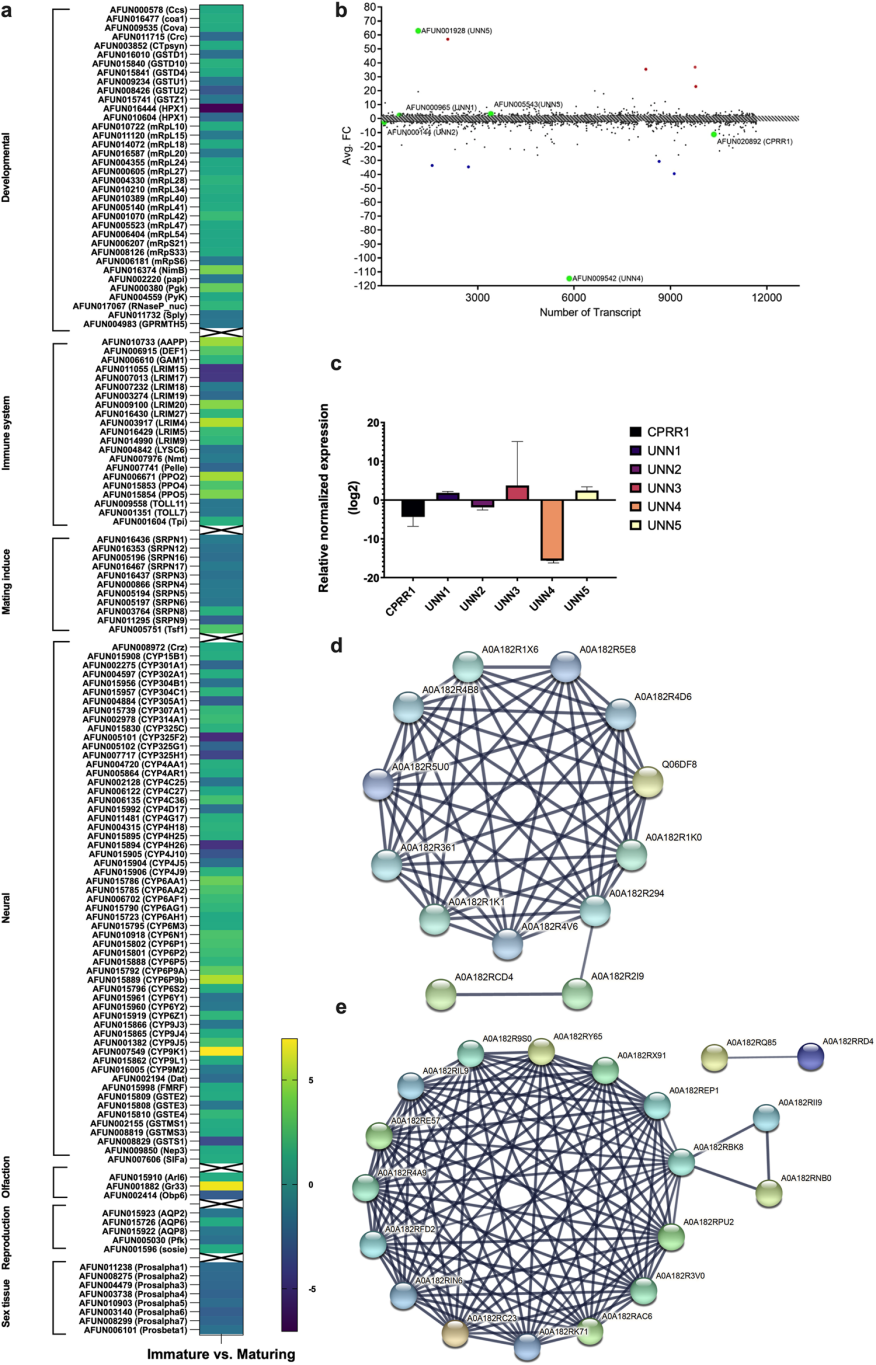
Transcript abundance in male *An. funestus* is affected by sexual maturation. (**a**) Male mosquito transcripts demonstrating reliable expression (> 1 TPM) in the whole body of immature (S0) and maturing (S3) were analysed in pairwise comparisons displayed as log_2_ fold change in abundance. Genes are arranged into gene families, developmental immune system, mating induce, neural, olfaction, reproduction, sex tissue (log_10_ TPM). Only transcripts which were significantly differenced in abundance (fold change > 2; FDR-corrected *p* < 0.05) are indicated in the heatmap. (**b**) it was shown a scatter plot of Avg. fold-change of differentially expressed genes, Y-axis depicts the average log2 fold-change, x-axis depicts the number of transcripts. Blue dots represent the top five under-expressed transcripts in maturing males compared to immature males, red dots depict the top five over-expressed transcripts in maturing males compared to immature males. The green dots represent the transcripts that were validated using qPCR. The grey bar indicates transcripts that did not exceed the fold-change cut of 2 ≤|FC|≤ − 2. (**c**) Validation of the normalized expression of RNA-Seq transcripts. The bars indicate the expression of the transcripts of males with genitalia rotation at S3 compared to S0. The transcripts were relative to the control group (S0), which is set to zero. The y-axis depicts the relative normalized expression in log2. The results were normalized to the reference genes *RPL19* and *RPS26*. *CPRR1* (AFUN020892), *UNN2* (AFUN 000144) and *UNN4* (AFUN009542) of males with genitalia rotation at S3 were down regulated compared to S0 of their corresponding transcript. *UNN1* (AFUN000965), *UNN3* (AFUN005543) and *UNN5* (AFUN001982) of males with genitalia rotation at S3 were up regulated when compared to S0 of their corresponding transcript. All samples *P* value ≤ 0.05. (**d**–**e**) are shown, uniprot ids for up- and down-regulated genes based on differentially expressed transcripts between Immature and mature cohorts (FC). Up and down figures show PPI networks extracted for up and down-regulated genes using the following parameters: max number of interactors at 1st shell:10; minimum required interaction score: 0.70; active interaction sources: all except the text mining one. (**d**) String protein annotation for up-regulated transcripts and (**e**) string protein annotation for down-regulated transcripts. String interactions of up-regulated transcripts and string interactions of down-regulated transcripts contain links between proteins at each network.

The ‘neural group’ contained the largest number of DEGs and interestingly, a large number of monooxygenase transcripts grouped under the neural category increased in transcript abundance during maturation (Fig. 3a). These include *CYP302A1* (1.5-fold increase), *CYP6P9a* (3.3-fold increase), *CYP6P9b* (4.4-fold increase) and *CYP9K1* (almost 6-fold increase) in maturing males. In addition, monooxygenase, *GSTS1-1* was 3.4-fold more abundant in the immature males. These enzymes have been associated with detoxification of insecticides. Other neural transcripts that were significantly differentially expressed between immature and maturing males included a neuropeptide, SIFamide (SIFa) and endopeptidases, Neprilysin- 3 (Nep3).

A total of 21 immune-related transcripts were also differentially expressed during the maturation process. These include leucine-rich repeat immune (*LRIM*) protein family (LRIM 4, 5, 9, 15, 17 and 19) amongst others. Five transcripts were statistically significantly differentially expressed (*p* < 0.05) during maturation and mainly related to reproduction process in various organisms, which are grouped under the ‘reproduction’ category in this study as well. For example, aquaporins (*AQPs*) are the well-studied transcripts in this group, a family of channel proteins that facilitate the transport of water and small solutes across biological membranes. They are widely distributed throughout the organism, having several key functions, some of them unexpected, both in health and disease. AQPs are involved and regulated in specific mechanisms underlying infertility in mammals^48,49^.

An ATP-dependant 6-phospfructokinase (*Pfk*) also called PKFM showed 2-fold abundance in the immature adults compared to the maturing males. This is a regulatory enzyme of the glycolysis pathway, important in regulating cells energy requirements. This enzyme breaks down fructose-6-phosphate to fructose 1,6-bisphosphate. A novel gene called *Sosie* (*sie*) known to be involved in various morphogenesis processes in *Drosophila* oogenesis fell within this category and was abundantly expressed in maturing males (Fig. 3a). S*osie* is also involved in the maintenance of structures of the actin cytoskeletons during oogenesis^50^.

The ‘olfaction’ category only identified three statistically significant DEGs associated mainly with the chemosensory ability, as expected. These include an odorant-binding protein 6 (*Obp6*) transcript, that showed an almost threefold abundance in the immature males compared to the maturing males. Another transcript ADP-ribosylation factor-like protein 6 (*Arl 6*), known to be present in male antennae only^51^, was more abundant in the tissue of the maturing males. Male-based transcript, *Gr33* (gustatory chemosensory receptor) increased in abundance during maturation. Matured males of both *An. coluzzii* and *An. quadriannulatus* express the gustatory receptor *Gr33* in their antennae at high levels, whereas this receptor is absent in female antennae^43,52^. We speculate that it plays a role in mating behaviour, either during swarm formation, cuticular carbohydrate tasting or recognition of conspecific females. Furthermore, eight transcripts associated with “sex tissue” showed a statistically significant difference (*p* < 0.05) in abundance between the immature and maturing males. As expected, these were sperm individualization proteins and specifically protease enzymes (prosalpha 1, 2, 3, 4, 5 and 6) as well as the proteasome subunit beta (prosbeta1) known to be involved in spermatogenesis.

Due to the role of the arthrodial membrane during genital rotation, analysis revealed that there was a total of 35 DEGs annotated to various cuticular families (2 ≤|FC|≤ − 2) between the immature and maturing males (Supplementary Table 2). Thirty-two of these were more abundant in the immature males and only three transcripts showed higher abundance in the maturing males. There were 10 transcripts abundant in immature males (|FC|≤ − 2) that were annotated to the cuticular RR-1 family. Arthropod cuticles contain many structural proteins, along with chitin, from diverse families. However, it is not very clear how these different cuticular proteins contribute to the overall structure of the cuticle. The RR-1 and RR-2 proteins are located in different regions of the cuticle. The RR-2 proteins are found in the exocuticle, which becomes sclerotized, while the RR-1 s are located in the endocuticle, which remains soft. Only two transcripts part of cuticular RR-1 family were abundant (|FC|≥ 2) in maturing males. Other cuticular families included the cuticular RR-2 family, where there were10 DEGs abundant in immature males. The abundance of other cuticular families such as the ‘fifty-one amino acid family’ (n = 3), CPLCG family (n = 2), CPFL (n = 1), CPCFC (n = 1), CPLCA (n = 1), as well as four transcripts that have not been classified into a family, were also more abundant in the immature males (Supplementary Table 2).

### Validation of selected expressed genes detected by RNA-seq

Six transcripts were selected for validation using qPCR (Fig. 3b and Supplementary Table 3). The purpose of the qPCR was to validate the RNA-seq dataset. As this was done on whole-body tissue, the qPCR was conducted on whole body tissue as well. The purpose was not to confirm the biological role and tissue location of the differentially expressed transcripts. Three of these were abundant in whole-body tissue of immature males and included *CPRR1* (AFUN020892) with a 11.39-fold difference, and *UNN2* (AFUN000144) with a 2.5-fold difference. The final transcript, *UNN4* (AFUN009452), was 114.72-fold more abundant in immature males when compared to maturing males. Both *UNN2* and *UNN4* were unannotated when the study was completed. The *CPRR1* (AFUN020892) transcript was grouped under the ‘immune system’ (Supplementary Fig. 2). The other three transcripts that were abundant in the whole-body tissue of maturing males were all unannotated. The *UNN1* (AFUN000965), *UNN 3* (AFUN005543) and *UNN5* (AFUN001928) showed a 2.403, 3.43 and 62.94-fold abundance respectively in maturing males. *UNN1* (AFUN000965) were also grouped under the ‘immune system’ as per Supplementary Fig. 2.

The normalized expression of the qPCR results (Fig. 3c; Supplementary Table 3), validated the RNA-Seq results. The abundance of RNA-Seq DEG in immature males was confirmed by qPCR as well as those abundant in maturing males. *CPRR1* (AFUN020892), *UNN2* (AFUN000144) and *UNN4* (AFUN009542) were all down-regulated in maturing males compared to immature males by a log 2-fold change of − 4.339, − 1.872 and − 15.610 RNE (relative normalized expression), respectively. Whereas *UNN1* (AFUN000965), *UNN3* (AFUN005543) and *UNN5*(AFUN001928) were all up-regulated in maturing males compared to immature males by a log 2-fold change of 1.864, 3.755 and 2.461 RNE, respectively (Fig. 3c; Supplementary Table 2). *CPRR1* (AFUN020892), *UNN1* (AFUN000965), *UNN2* (AFUN000144), *UNN3* (AFUN005543), *UNN4* (AFUN009542) and *UNN5* (AFUN001928) all had a differential RNE with a *p* value threshold of < 0.05, indicating that the differential expression of the selected transcripts was statistically significantly different between the immature and maturing males (Fig. 3c; Supplementary Table 3).

### Network analysis and visualization of sexual maturation data

We further analyzed the potential interaction between these two male developmental stages using STRING software for the prediction of protein–protein interaction. The functional impacts of DEGs were surveyed in system analysis with PPI network construction (Fig. 3d–e). The constructed network was consisting of an interaction score set on > 0.7 (high confidence). The generated network was clustered into two modules. The first module, which focused on up-regulated genes, showed an interaction among all biological processes except developmental genes, which branched as a separate group of genes. The second module showed down-regulated gene interactions as three different interactive groups with the genes also showing intense interaction. However, neural genes and immune-related genes announced into separated networks. The immune network presented a connection to the main gene network via *Cactus*, (a negative regulator of *REL1*), which has an effect on mosquito innate immunity by decreased pathogen susceptibility and low levels of *REL1* gene expression. The Toll pathway can regulate the expression of several antimicrobial peptides in mosquitoes. Uncharacterised genes also showed a close connection with immune-related genes. Additionally, it was demonstrated that the Toll pathway activity differs between male and female *D. melanogaster* in response to bacterial infection, thus identifying innate immune signalling as a determinant of sexual immune dimorphism^53^. The main pathways in which each node of the networks is involved are reported in the table biological process for up- and down-regulated genes (Supplementary Tables 4–6).

## Discussion

This is the first transcriptomic study examining the maturation in male *Anopheles funestus* with the aim of providing some insight into their sexual maturation and aging post-eclosion. In addition, this study provided a driven network among transcripts identified during male ageing (Supplementary Fig. 3). This study identified a large number of unannotated genes, which emphasises the importance of further study on the gene products of this neglected species.

The biological importance of the male *An. funestus* has been neglected since they are not directly involved in disease transmission^54,55^. This has gradually changed as their potential as a target for vector control has been recognized^56^. The adult male mosquitoes’ main biological role is currently assumed to be mating^19^. Disruption of the mating process will directly impact the population’s reproductive success; however, the lack of knowledge on the molecular mechanisms involved in male sexual maturation is limiting research expansion in this field. The physiological process of sexual maturation is complex and we used the rotation of the genitalia as a physiological marker to differentiate between immature adult males, with genitalia rotation at S0, and sexually maturing adult males, with genitalia rotation at S3. RNA sequencing revealed a large number of DEGs (n = 455) between these two genitalia rotation stages when using whole-body tissue. The immature males showed a higher transcript abundance compared to the maturing males. The level 3 gene ontology analysis revealed that a large number of these genes were functionally unannotated and limited the interpretation of the full data set. However, results identified a large number of genes associated with transcription/translation activities in the males of this major malaria vector species.

Sexual development in mosquitoes is regulated by the central and peripheral nervous system. During male mosquito sexual maturity, there was a highly-maintained regulation of the nervous system transcription. Male genitalia rotation and sexual maturity are regulated by the nervous system, with more transcripts for the central nervous system present in the immature stage (S0). As the organism matures (S3), there is a shift towards the peripheral nervous system. Interestingly, our results showed that the proportion of differentially-abundant transcripts for the central nervous system, including cell communication, visual perception, and sensory perception of sounds, was higher in immature stage (S0) than maturing stage (S3). Notably, there was a significant pronounced shift towards the peripheral nervous system in maturing stage (S3,) such as the chemosensory receptor, photoreceptor and taste receptor activities. Furthermore, the study also identified many monooxygenases that were differentially expressed in immature and maturing males. This includes *CYP302A1*, a P450 gene that encodes the JH enzyme (22-hydroxylase), which is a vital enzyme in the ecdysone biosynthesis pathway^57^. In *Drosophila* this gene is a mitochondrial P450^57,58^. The expression of this gene was present in the MAGs as well as in the fat body of *B. dorsalis*^38^.

Additionally, P450s associated with pyrethroid resistance in this species were also identified as expected and include *CYP6P9a* (previously called *CYP6P9*) and *CYP6P9b* (previously called *CYP6P13*). Both these genes have been identified as major detoxification enzymes in pyrethroid-resistant *An. funestus*^3,59,60^. Similarly, *CYP9K1* has also been associated with pyrethroid resistance in *An. funestus* from eastern Uganda^61^ as well as in deltamethrin resistance in *An. gambiae*^62^. These P450s associated with pyrethroid resistance were more abundant in maturing adults. This is not unexpected, as newly-emerged adults are more susceptible to insecticides, and therefore adults older than three days are generally evaluated when resistance detection assays are performed^63^. Other P450s showed higher abundance in the immature adults and include *CYP305A1*, *CYP325F2*, *CYP325H1*, *CYP304B1* and *CYP325G1*, to mention a few. In addition to P450s, glutathione S-transferases (*GSTs*) were also found to be abundant in immature males. Although *GSTS1-1* was identified to be upregulated in pyrethroid-resistant male *An. funestus*^60^, the abundance was reduced in maturing males. It will be important to clarify the importance of these enzymes in the maturation process of male *An. funestus* mosquitoes.

A large number of immune transcripts were differentially expressed in the immature and the maturing males; however, it is difficult to predict if they are specific to the sexual maturation process. Maturing males will most likely have ingested sugar water for a longer period of time compared to the immature males, and this might have initiated a response against any bacteria ingested. This might explain the increase in transcript abundance in the immune genes observed in maturing males, but further studies are needed to confirm this hypothesis. However, some of these transcripts were more abundant during immature stages and it is difficult to predict if these provided protection during the adult emergence or pupal stage, and future research on these DEGs might provide detailed information on the male immune system. Furthermore, Wei et al.^38^ indicted that immune genes might play an important role in the male reproductive tract to protect against pathogens that might have deleterious effects after or during mating. A large body of research exists on the immune interaction in the female when challenged with pathogens, but even this process is poorly understood^64–66^.

The identification of 10 *CPRR-1* DEGs was encouraging and warrants further investigation. Although these genes are associated with the arthrodial membrane, it will be important to identify the localisation of these proteins within the male body. These membranes might be vital for movement during the genital rotation process, but also for flight or movement of other appendages e.g. legs. It was surprising that this study did not identify any myosin superfamily of actin-based motor proteins involved in muscle contraction and ATPase activity^21^ differentially expressed, even though this family of proteins are important during *Drosophila* genitalia rotation^23^. Using whole-body tissue might have masked DEGs like these, and this is a limitation of the current study. Future work should investigate its importance using tissue-specific experiments.

It was reassuring that this study was able to identify previously-published male-based genes known to be in male antennae. *OBP6* was however more abundant in the immature stage, while *Arl 6* and *Gr33* were more abundant in the maturing adults. Wang et al.^67^ recently found that moths with an increase of *OBP6* expression, showed an increased flight ability. *Gr33* is a chemosensory receptor and might be a critical receptor in males for responding to swarming pheromones during swarming activity. However, the published information on these genes remains limited and as above, it is essential that future, more tissue-specific studies be conducted to understand the molecular expression profile of these target genes. Furthermore, it's possible that future studies on *An. funestus* could improve our understanding of gene roles and functions if the dataset is compared to orthologous genes in a better annotated anopheline species like *An. gambiae*. This is because the current annotation for *An. funestus* is limited and could benefit from comparison to a more well-annotated species.

Selected DEGs analysed using qPCR to validate the data set and direction of expression (abundance within a specific stage) were confirmed by all six transcripts, indicating that the DEGs identified in this study provide additional information for future follow-up research. Our findings present only the tip of the iceberg of neuro-immune plasticity during male mosquitoes’ short life and suggest the involvement of network between male aging and sexual development. This study also provides a firm basis for future investigation to understand the function of these transcripts and manipulating them to interrupt or prevent mating and reproduction.

## Conclusion

The limited genomic information on *Anopheles funestus* was challenging for this study, and highlights the importance of improving whole genome sequencing information. Further functional genomics on this species is vital to increase our understanding of this important African malaria vector. We provide new insights into the interplay between genetics and physiology in male mosquitoes. This information will reshape our understanding of this insect, as to date communal activity (or group decision-making) has not been demonstrated in the vector control field. Therefore, the outcomes will directly support the development of vector-control strategies/methods, such as gene‐drive and vector surveillance monitoring.

## Materials and methods

### Biological material

*Anopheles funestus* males were reared and housed in standard insectary (Botha De Meillon insectary at the NICD, NHLS, Sandringham, Johannesburg, South Africa) conditions with a 12 h light/dark cycle at 25 ± 2 °C and 80 ± 10% humidity. *Anopheles funestus s.s.* males (FUMOZ colony) originating from southern Mozambique in 2000 were used in this study^68^.

### Sample preparation

It was hypothesized that the sexual maturation process will be complete in *An. funestus* males once their genital rotation had completed at stage 4 (S4), and that some transcripts associated with maturation might no longer be present or only present at low levels. Hence transcript comparisons were evaluated between young adults (0–2 h post-eclosure) without genital rotation called ‘immature’ (for this study) and 14–16 h old males with a 90–135° genital rotation (referred to as ‘maturing’ in this study). Newly-emerged males were collected within two hours after emergence and placed in rearing cages (30 × 30 × 30 cm BugDorm® insect cages (Megaview Science Education Services Co Ltd, Taiwan). Adult *An. funestus* males were aged up to two hours to represent the young immature males (S0); after an additional 14 h males were nearly mature or S3 adults. Prior to RNA extraction, the S0 or S3 genital rotation was confirmed by microscopy^8^. Males were collected on three separate days to represent three biological replications.

### RNA extraction and preparation

Total RNA was extracted for each biological replicate using ten male *An. funestus* mosquitoes at the specified age (immature [S0 genitalia rotation] or maturing [S3 genitalia rotation]) (equivalent to 100 mg of biological material) using Life Technologies TRIzol®Reagent (CAT. No.: 15596). The RNA concentration was measured in ng/µl using a NanoDrop 2000 spectrophotometer. The quality and integrity of the RNA were evaluated by electrophoresing the RNA on a native agarose gel^69^. The resulting RNA was also quality and quantity checked by using Agilent Technologies 2100 bioanalyzer (or 2200 TapeStation). Samples with a final nucleic acid concentration > 260 ng/µL (260/280 ratio > 2) and a relative integrity number (RIN value) above 6 were used.

### RNA sequencing

Macrogen Inc. performed RNA sequencing (RNA-Seq) on *An. funestus* males, immature as well as maturing, using the Illumina HiSeq2000/2500. Macrogen Inc. eliminated DNA contamination using DNase and performing poly-A selection, followed by randomly fragmenting the purified RNA for short-read sequencing^70^. Selection of 3′ poly-A tails ensured that only mature RNA representing coding sequences was selected^70^. The fragmented RNA was reverse transcribed into cDNA and adaptors were ligated on both ends of the cDNA fragments to allow for PCR amplification^70^. Fragments with insert sizes between 200 and 400 bp were selected for paired-end sequencing. Both ends of the cDNA were sequenced by read length. The data analysis was performed on the sequences to set up expression profiles and functional annotation reports. The raw-read sequences were deposited in the Sequence Read Archive (SRA) and can be viewed and downloaded under the identifier SRP246051 (https://trace.ncbi.nlm.nih.gov/Traces/sra/?study=SRP246051).

### Bioinformatic analysis

The FastQ files from the sequencing were evaluated by Macrogen Inc. for overall quality using FastQC. Adapter sequences and low-quality reads were removed using Trimmomatic (version 0.36; parameters: ILLUMINACLIP:TruSeq3-PE.fa:2:30:10 SLIDINGWINDOW:4:15 MINLEN:70). Reads retained after quality control were mapped to the mosquito reference genome using HISAT2 (version 2.1.0, using default parameters). Reference genome sequence and annotation were downloaded from Vectorbase.org (FUMOZ, version-01-2020). Gene read counts were generated using HTSeq-count (version 0.9.1; parameters: -s no -t exon -i gene_id -r pos -m intersection-nonempty) and custom bash scripts (available on demand). Differential gene expression analysis was performed in R using DESeq2 package with default parameters. Visualisations were performed using various graphical packages in R and GraphPad Prism.

### Gene ontology analysis

The PANTHER (protein annotation through evolutionary relationship) classification system was used to identify *An. funestus* biological processes enriched in each stage cluster and in the group of differentially expressed transcripts that show enrichment. The significance of the statistical overrepresentation test was analysed by applying Fisher’s Exact test with False Discovery Rate correction. The raw read sequences were quality controlled by FastQC v0.10.0, where the overall read quality; total bases, total reads, GC (%) and basic statistics were calculated^71^. A phred quality score of Q20 (%) and Q30 (%) were accepted as good quality^71^. Low-quality, adaptor sequences, contaminant DNA or PCR duplicates were removed from the analysis to reduce bias. To analyze the functions of DEGs, GO enrichment of DEGs was performed using the STRING database in three biological processes, molecular function and cellular component categories^72^. Then FDR correction was used to account for multiple testing (*p* adjust value cutoff < 0.05).

### Quantitative real-time PCR (qRT-PCR) validation

One selected transcript with known biological process and molecular function as well as three unannotated transcripts were used to validate sequencing results with qRT-PCR. These were selected based on similar differential expression levels (≥ 2-fold change) across all three biological replicates from the transcriptome sequencing data set. The transcripts selected for validation were the AFUN020892 (a CPRR-1 endocuticle family transcript), and three un-annotated DEG transcripts (UNN1 [AFUN000965], UNN2 [AFUN000144] and UNN3 [AFUN005543]). The UNN4 (AFUN009542) and UNN5 (AFUN001928) transcripts were selected as they had the lowest and the highest average |FC|, across the three biological replicates, respectively (www.VectorBase.org un-annotated accessed on 3 June 2020). cDNA was then reverse transcribed from RNA of immature and maturing *An. funestus* males using the Qiagen QuantiTect Reverse Transcription kit^73^. Primers were designed by using the Beacon Designer ™ (Premier Biosoft) or Invitrogens free online primer design tool, OligoPerfect™ Designer) based on the sequence information of *An. funestus* transcripts*.* The relative expression of the chosen transcripts was normalized with two selected reference genes (RG) using the Bio-Rad CFX Maestro™ Software version 4.0.2325.0418^74^. The most stable reference genes were selected after evaluation of five possible reference genes^73^. The qPCR reaction (25 µL reactions) consisted of iQTM SYBR® Green Supermix (CAT: 170-8882) and the specific transcript primer pairs. For CPRR1 (AFUN020892; Forward: TCTCAAACCACGACATCTGC; Reverse: GTTGACCGTGACCAGAGGAT), UNN1 (AFUN000965; Forward: CGTACATCTGGGGTAACATAGTGG; Reverse: GCACTGTAACCTATCTCGTCCTG) UNN2 (AFUN000144; Forward: ATGTCCTCGGATGAGAGTGG; Reverse: GTCCCTGTTACGGTTGCATT) UNN3 (AFUN005543; Forward: AGGGGTTACCGTGATCAC; Reverse: GCACACGAGTTATCCACATG) UNN4 (AFUN009542; Forward: AGTGCCATCGAAGCGATCCA; Reverse: GTGGTTCCAGCGGCAAGATG) and UNN5 (AFUN001928; Forward: ACCCGTACGACGTTCTTCCC Reverse: CTCCTCCATACATGGCCGCA) in a concentration of 0.3 µM. The protocol was identical for all primer sets used (94 °C for 2 min; 39 X (94 °C for 30 secs, transcript specific annealing temperature for 30 s, 72 °C for 30 s) 72 °C 10 min), except for the annealing temperature, which was optimized during primer selection. CPPR1 primers used an annealing temperature of 63.3 °C and the UNN transcripts all had an annealing temperature of 61.4 °C. The samples were amplified in a Bio-Rad CFX96™ Real-Time System Optics module C1000 Touch Thermal Cycler in a 96-well plate. Three biological replicates as well as three technical replicates were used during the analysis. RPL19 and RPS26 were used as reference genes to normalize the data. The significance level (α) was set to 0.05. The *ΔΔcq* method was used for analysis using the Bio-Rad CFX Maestro software (2006).

### Supplementary Information

Supplementary Information.

## Data Availability

All data are available in the main text or the supplementary materials. The code for the network data analysis has been deposited in GitHub and is available under the https://github.com/Noushin-Emami/An-funestus-male.git. Any additional information required to reanalyse the data reported in this paper is available from the lead contact upon request.
