# Supplementary material for: Data-driven networking of global transcriptomics and male sexual development in the main malaria vector, Anopheles funestus
Source: Sci Rep. 2023 Oct 5;13:16798. doi: 10.1038/s41598-023-43914-0 (PMC10556010; doi:10.1038/s41598-023-43914-0)
Supplement: Supplementary file 1 — Supplementary Information. [file 41598_2023_43914_MOESM1_ESM.pdf]

Supplementary document

Data-driven networking of global transcriptomics and sexual development in main malaria vector, *Anopheles funestus*.

This PDF file includes:

Figures: Supplementary Fig. 1 to 3

Tables: Supplementary Table 1 to 6

Table 1: Raw data statistics of the RNA-sequencing data of each replicate.

**Sample I.D.***	**Total read bases (Gb)****		**Total reads**		**GC (%)**		**Q20 (%)**		**Q30 (%)**	
	**U**	**T**	**U**	**T**	**U**	**T**	**U**	**T**	**U**	**T**
**St. 0.1**	8.736	8.611	86,499,406	85,690,086	45.03	45.03	98.78	99.09	96.14	96.59
**St. 0.2**	10.235	10.050	101,343,778	100,020,088	43.81	43.81	98.57	98.99	95.66	96.25
**St. 0.3**	11.110	10.931	110,000,778	108,804,004	45.93	45.93	98.65	99.00	95.82	96.33
**St. 3.1**	9.906	9.753	98,084,094	97,088,078	42.40	42.38	98.67	99.00	95.78	96.25
**St. 3.2**	7.756	7.635	76,792,800	76,021,002	41.91	41.89	98.74	99.07	96.00	96.46
**St. 3.3**	11.482	11.314	113,686,858	112,595,782	43.85	43.84	98.74	99.06	95.98	96.44

*St. n.n = Stage n. biological replicate number n

U = Untrimmed data

T = Trimmed data

The total number of bases reads, GC (%), Q20(%) Q30(%), were calculated for the 6 samples. **Total read bases = Total reads X Read Length.

Q20(%): is the ratio of reads that have a phred quality score of over 20, Q30(%): is the ratio of reads that have a phred quality score of over 30.

Table 2: Number of cuticular transcripts that were differentially expressed (|FC| ≥2) and their families.

Cuticular family	Number of transcripts abundant in immature males	Number of transcripts up abundant in maturing males
Cuticular RR-1 family	10*	2
Cuticular RR-2 family	10	0
fifty-one aa family	3	0
CPLCG family	2	1
CPFL family	1	0
CPCFC family	1	0
CPLCA family	1	0
Unclassified family	4	0
**Total**	32	3

*Includes AFUN020892 (CPRR1) transcript.

Table 3: qPCR normalized expression results.

Target*	Relative Normalized Expression of qPCR (log2)	P-Value of qPCR RNE	Relative Normalized Expression of RNA-seq data (log2)
Control (S_0)	N/A	N/A	N/A
CPRR1 (S_3)	**-4.339**	**0,007234**	**-11.386**
UNN1 (S_3)	**1.864**	**0,000011**	**2.403**
UNN2 (S_3)	**-1.872**	**0,007364**	**-2.500**
UNN3 (S_3)	**3.755**	**0,000017**	**3.425**
UNN4 (S_3)	**-15.610**	**0.025277**	**-114.715**
UNN5 (S_3)	**2.461**	**0.031855**	**62.939**

Green = Up-regulation in expression

Blue = Down-regulation in expression

RNE = *Relative normalized expression*

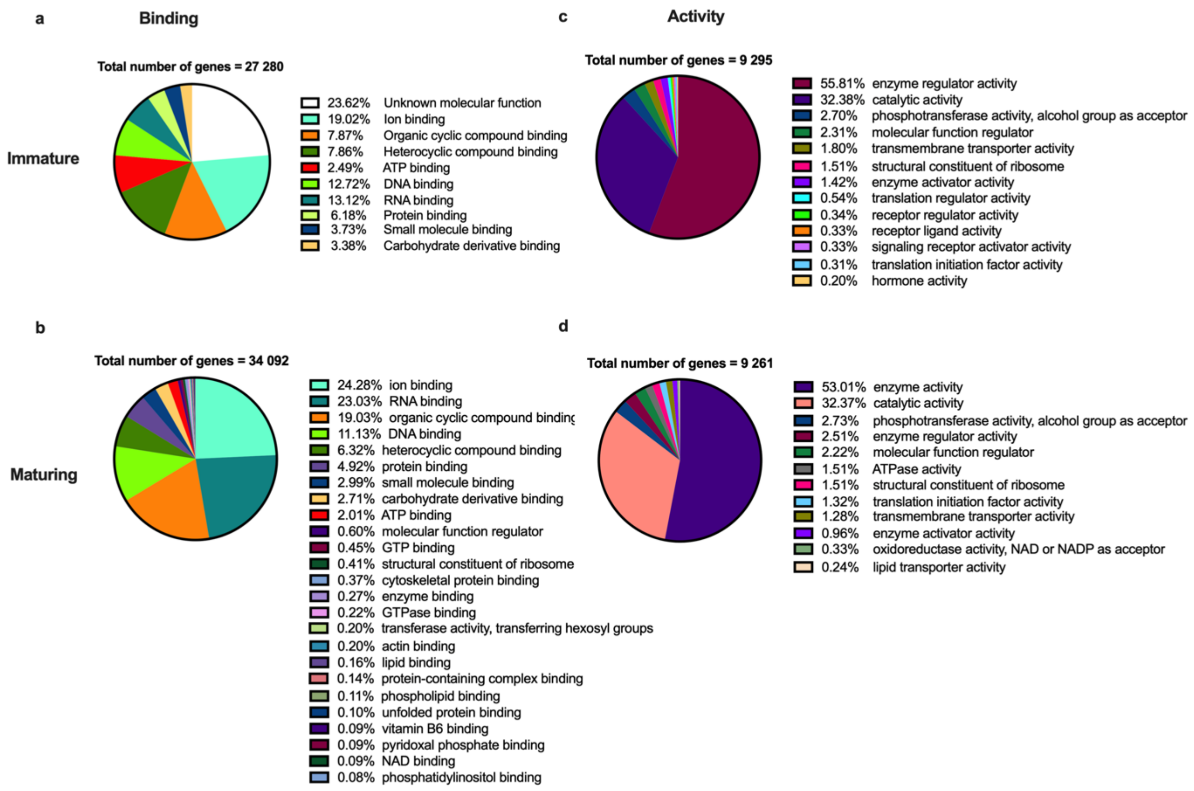

Supplementary Fig 1. A level 3 gene ontology analysis of molecular functions in the whole body of *An. funestus*. (a-b) The proportion of binding related genes that are reliably detected in Immature and Maturing males. (c-d) The proportion of activity related genes that are differentially abundant between Immature and Maturing males. The pie-charts generated with bioinformatics tools provided by SNIC through the Multidisciplinary Center for Advanced Computational Science (UPPMAX) under project SNIC 2021/22-492, https://supr.naiss.se/proposal/20544/ and organized using GraphPad Prism. *GraphPad Software version 6.04*, <http://www.graphpad.com/> (2021).

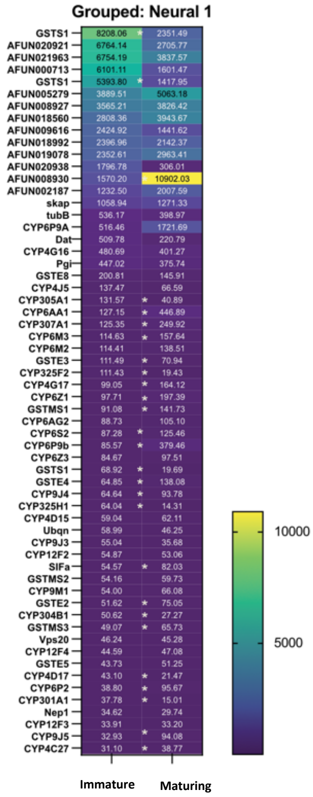
Grouped: Neural 1
Immature
Maturing
10000
5000

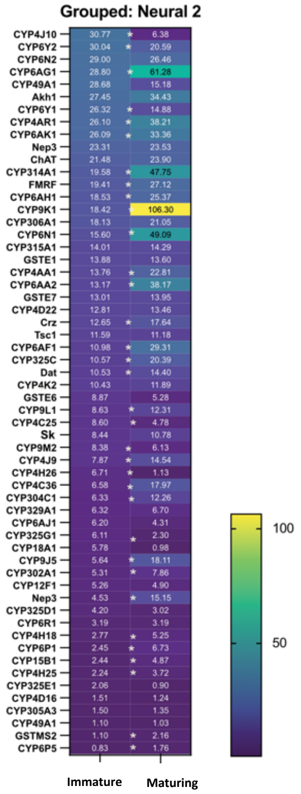
Grouped: Neural 2
Immature
Maturing
100
50

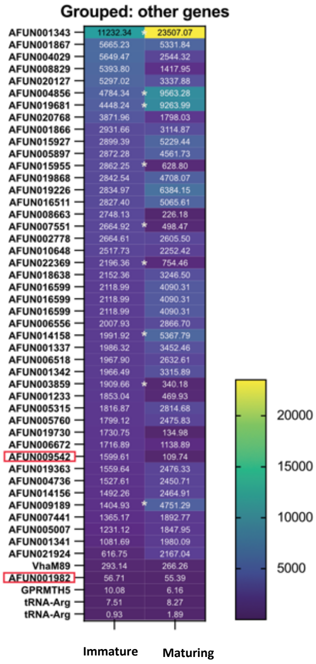
Grouped: other genes
Immature
Maturing
20000
15000
10000
5000

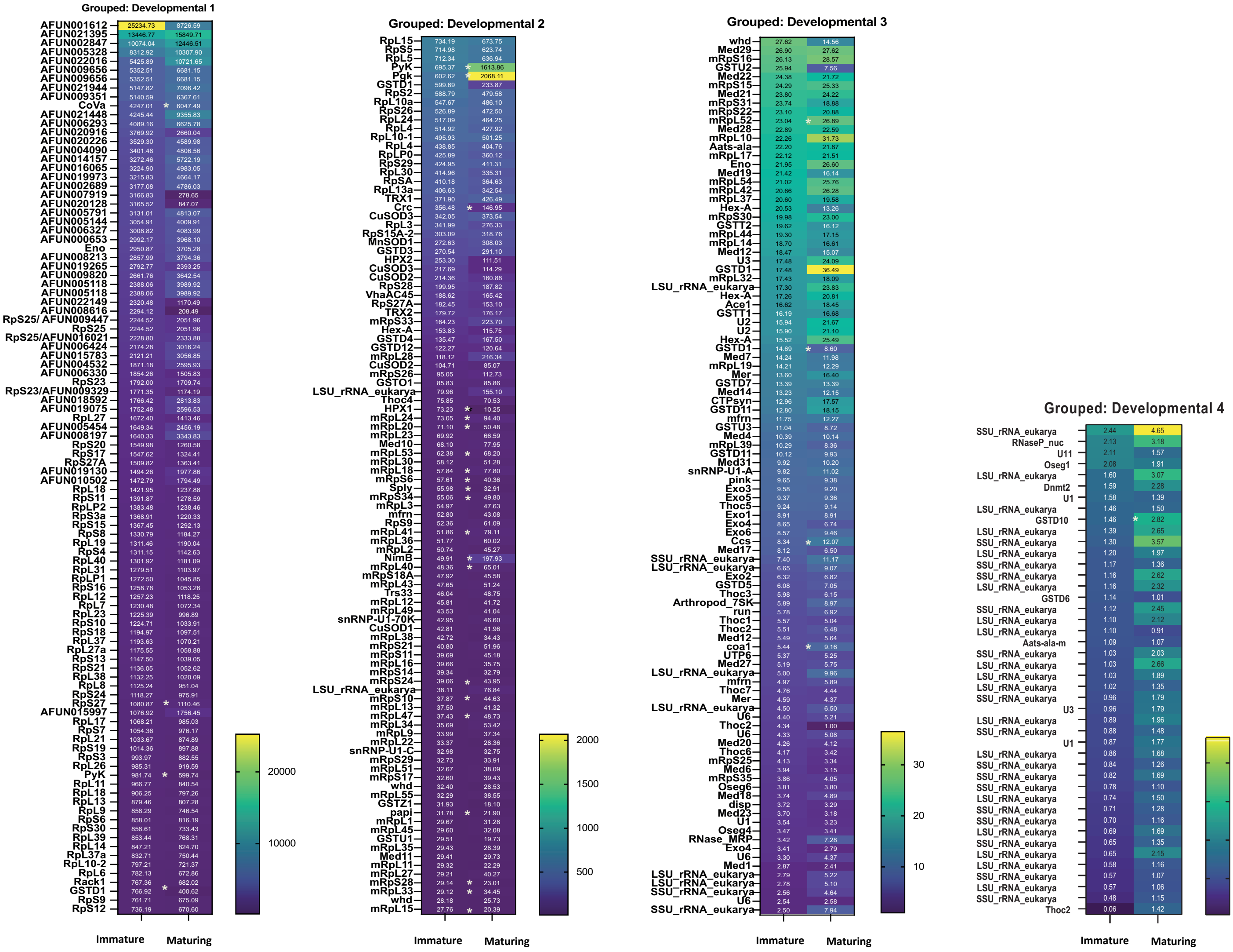
Grouped: Developmental 1
Grouped: Developmental 2
Grouped: Developmental 3
Grouped: Developmental 4
Immature
Maturing
20000
10000
2000
1500
1000
500
30
20
10
4
3
2
1

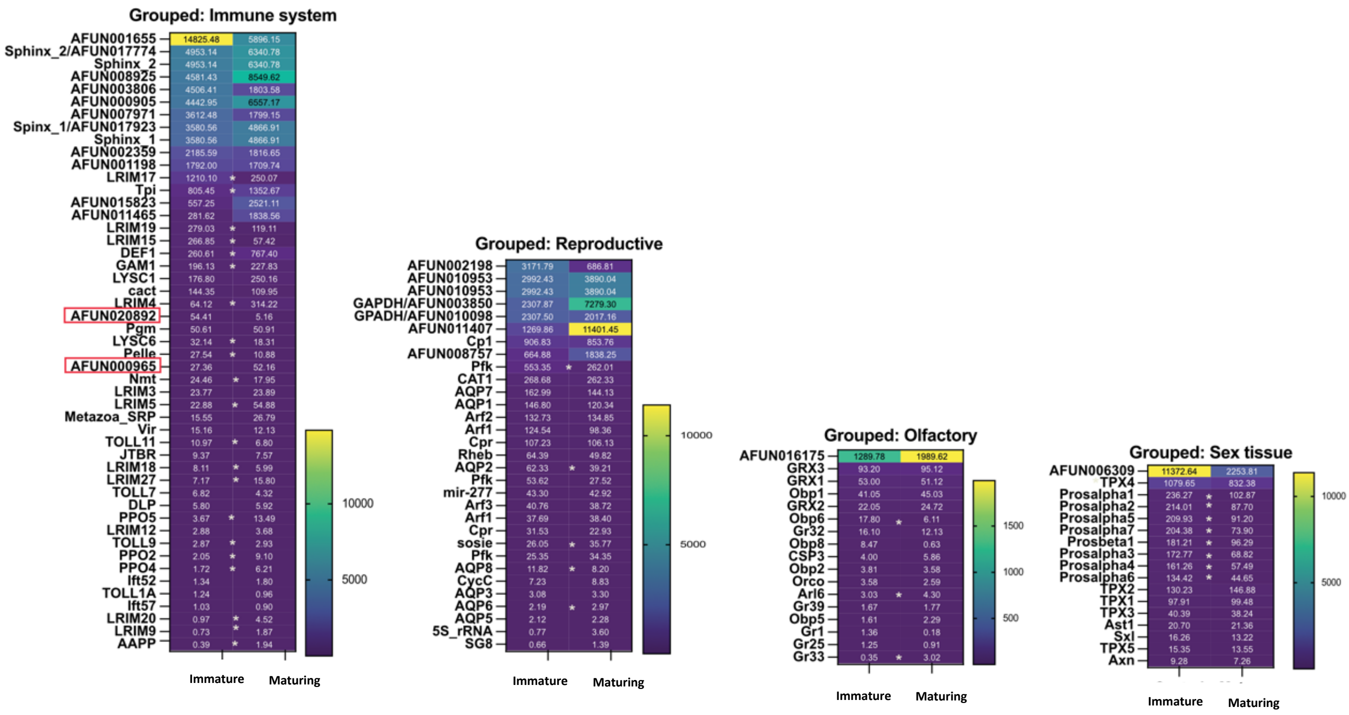

Supplementary Fig 2. Heatmaps showing the relative abundance and distribution of the *An. funestus* male mosquito transcripts in different categories including Neural, developmental, immune system, olfactory, sex tissue, and others within the global male mosquito tissues for the different cohort. S0, Immature and S3, Maturing. Candidate genes with a false-discovery rate (*p* value) less than 0.05 are shown by *. Heatmaps made by log2-transformed reads per kilobase per million reads (TPM) abundance values of mosquito genes identified from male sample pools. The heatmaps generated with bioinformatics tools provided by SNIC through the Multidisciplinary Center for Advanced Computational Science (UPPMAX) under project SNIC 2021/22-492, https://supr.naiss.se/proposal/20544/ and organized using GraphPad Prism. *GraphPad Software version 6.04*, <http://www.graphpad.com/> (2021).

Table 4: The main pathways are involved in the biological process for up regulated genes.

#node	identifier	domain_summary_url
A0A182R1K0	62324.A0A182R1K0	40S ribosomal protein S3; Belongs to the universal ribosomal protein uS3 family.
A0A182R1K1	62324.A0A182R1K1	60S ribosomal protein L6
A0A182R1X6	62324.A0A182R1X6	60S ribosomal protein L21
A0A182R294	62324.A0A182R294	60S ribosomal protein L8
A0A182R2I9	62324.A0A182R2I9	Phosphoglycerate kinase
A0A182R361	62324.A0A182R361	60S ribosomal protein L26
A0A182R4B8	62324.A0A182R4B8	60S ribosomal protein L27a
A0A182R4D6	62324.A0A182R4D6	60S ribosomal protein L27; Belongs to the eukaryotic ribosomal protein eL27 family.
A0A182R4H8	62324.A0A182R4H8	39S ribosomal protein L42, mitochondrial
A0A182R4V6	62324.A0A182R4V6	40S ribosomal protein S23; Belongs to the universal ribosomal protein uS12 family.
A0A182R5E8	62324.A0A182R5E8	60S ribosomal protein L18a; Belongs to the eukaryotic ribosomal protein eL20 family.
A0A182R5U0	62324.A0A182R5U0	40S ribosomal protein S2; Belongs to the universal ribosomal protein uS5 family.
A0A182R6B0	62324.A0A182R6B0	Uncharacterized protein
A0A182RCD4	62324.A0A182RCD4	Glyceraldehyde-3-phosphate dehydrogenase
A0A182RCJ8	62324.A0A182RCJ8	Leucine-rich immune protein (Long)
A0A182RHN9	62324.A0A182RHN9	Transferrin; Transferrins are iron binding transport proteins which bind Fe(3+) ion in association with the binding of an anion, usually bicarbonate.
A0A182RIS0	62324.A0A182RIS0	Cytochrome P450; Belongs to the cytochrome P450 family.
A0A182RK42	62324.A0A182RK42	Gambicin
A0A182RKA3	62324.A0A182RKA3	Prophenoloxidase 2
A0A182RKD4	62324.A0A182RKD4	Cytochrome P450; Belongs to the cytochrome P450 family.
A0A182RKZ7	62324.A0A182RKZ7	Defensin anti-microbial peptide
A0A182RMS7	62324.A0A182RMS7	Cytochrome P450; Belongs to the cytochrome P450 family.
A0A182RMX0	62324.A0A182RMX0	Uncharacterized protein
A0A182RW11	62324.A0A182RW11	Cytochrome b5 heme-binding domain-containing protein; Belongs to the cytochrome b5 family.
A0A182RW78	62324.A0A182RW78	Uncharacterized protein
A0A182RWF1	62324.A0A182RWF1	Sodium/hydrogen exchanger; Belongs to the monovalent cation:proton antiporter 1 (CPA1) transporter (TC 2.A.36) family.
A0A182RXA4	62324.A0A182RXA4	Cytochrome P450; Belongs to the cytochrome P450 family.
A0A182S031	62324.A0A182S031	DUF4371 domain-containing protein
A0A182S4L5	62324.A0A182S4L5	Cytochrome P450; Belongs to the cytochrome P450 family.
A0A182S4N6	62324.A0A182S4N6	Cytochrome P450; Belongs to the cytochrome P450 family.
A0A182S4R9	62324.A0A182S4R9	Cytochrome P450; Belongs to the cytochrome P450 family.
A0A182S4T9	62324.A0A182S4T9	Glutathione S-transferase
A0A182S4Y3	62324.A0A182S4Y3	Prophenoloxidase 4
A0A182S550	62324.A0A182S550	Cytochrome P450; Belongs to the cytochrome P450 family.
A0A1Y9HDQ3	62324.A0A1Y9HDQ3	Nimrod B2
A0A1Y9HDU8	62324.A0A1Y9HDU8	Leucine-rich immune protein (Short)
A0A1Y9HDV4	62324.A0A1Y9HDV4	Leucine rich immune protein (Coil-less)
A0A4Y0AR66	62324.A0A4Y0AR66	Cytochrome P450; Belongs to the cytochrome P450 family.
CYP6AA2	62324.B5AIH8	Cytochrome P450; Belongs to the cytochrome P450 family.
CYP6AA4	62324.B5AIH9	Cytochrome P450; Belongs to the cytochrome P450 family.
CYP6P1	62324.B5AII1	Cytochrome P450; Belongs to the cytochrome P450 family.
CYP6P9a	62324.B5AII6	Cytochrome P450 CYP6P9a; Belongs to the cytochrome P450 family.
CYP6P9b	62324.B5AII7	Cytochrome P450 CYP6P9b; Belongs to the cytochrome P450 family.
Ciao1	62324.A0A182RMX1	Probable cytosolic iron-sulfur protein assembly protein Ciao1; Essential component of the cytosolic iron-sulfur (Fe/S) protein assembly machinery. Required for the maturation of extramitochondrial Fe/S proteins.
Q06DF8	62324.Q06DF8	40S ribosomal protein S28

Table 5: The main pathways are involved in the biological process for down regulated genes.

#node	identifier	domain_summary_url
**A0A182R3V0**	62324.A0A182R3V0	This complex plays a key role in the maintenance of protein homeostasis by removing misfolded or damaged proteins, which could impair cellular functions, and by removing proteins whose functions are no longer required.
**A0A182R4A9**	62324.A0A182R4A9	Proteasome subunit beta
**A0A182R7P4**	62324.A0A182R7P4	Dopamine N-acetyltransferase
**A0A182R7X5**	62324.A0A182R7X5	Cytochrome P450
**A0A182R8B2**	62324.A0A182R8B2	Odorant-binding protein 6
**A0A182R9S0**	62324.A0A182R9S0	PCI domain-containing protein
**A0A182RAC6**	62324.A0A182RAC6	The proteasome is a multicatalytic proteinase complex which is characterized by its ability to cleave peptides with Arg, Phe, Tyr, Leu, and Glu adjacent to the leaving group at neutral or slightly basic pH; Belongs to the peptidase T1A family.
**A0A182RBK8**	62324.A0A182RBK8	Protein cactus (TOLL pathway signalling)
**A0A182RC23**	62324.A0A182RC23	The proteasome is a multicatalytic proteinase complex which is characterized by its ability to cleave peptides with Arg, Phe, Tyr, Leu, and Glu adjacent to the leaving group at neutral or slightly basic pH; Belongs to the peptidase T1A family.
**A0A182RE57**	62324.A0A182RE57	The proteasome is a multicatalytic proteinase complex which is characterized by its ability to cleave peptides with Arg, Phe, Tyr, Leu, and Glu adjacent to the leaving group at neutral or slightly basic pH; Belongs to the peptidase T1A family.
**A0A182REP1**	62324.A0A182REP1	Uncharacterized protein
**A0A182RFA6**	62324.A0A182RFA6	Cytochrome P450; Belongs to the cytochrome P450 family.
**A0A182RFD2**	62324.A0A182RFD2	Proteasome subunit beta
**A0A182RFW9**	62324.A0A182RFW9	Cytochrome P450; Belongs to the cytochrome P450 family.
**A0A182RFX0**	62324.A0A182RFX0	Cytochrome P450; Belongs to the cytochrome P450 family.
**A0A182RFX1**	62324.A0A182RFX1	Cytochrome P450; Belongs to the cytochrome P450 family.
**A0A182RG59**	62324.A0A182RG59	Serine protease inhibitor (serpin) 16; Belongs to the serpin family.
**A0A182RII9**	62324.A0A182RII9	Uncharacterized protein
**A0A182RIL9**	62324.A0A182RIL9	Proteasome subunit beta
**A0A182RIN6**	62324.A0A182RIN6	Proteasome subunit beta
**A0A182RK71**	62324.A0A182RK71	MPN domain-containing protein
**A0A182RKU1**	62324.A0A182RKU1	Cytochrome P450; Belongs to the cytochrome P450 family.
**A0A182RL95**	62324.A0A182RL95	Leucine rich immune protein (Coil-less)
**A0A182RN86**	62324.A0A182RN86	Cytochrome P450; Belongs to the cytochrome P450 family.
**A0A182RNB0**	62324.A0A182RNB0	TOLL pathway signalling Ser/Thr kinase
**A0A182RPU2**	62324.A0A182RPU2	Belongs to the peptidase T1A family.
**A0A182RQ85**	62324.A0A182RQ85	Glutathione S-transferase
**A0A182RRD4**	62324.A0A182RRD4	Glutathione S-transferase; Belongs to the GST superfamily.
**A0A182RWE4**	62324.A0A182RWE4	Heme peroxidase 2
**A0A182RX91**	62324.A0A182RX91	The proteasome is a multicatalytic proteinase complex which is characterized by its ability to cleave peptides with Arg, Phe, Tyr, Leu, and Glu adjacent to the leaving group at neutral or slightly basic pH
**A0A182RXN7**	62324.A0A182RXN7	Leucine rich immune protein (TM)
**A0A182RXP6**	62324.A0A182RXP6	Serine protease inhibitor (serpin) 19; Belongs to the serpin family.
**A0A182RY65**	62324.A0A182RY65	Proteasome subunit alpha type
**A0A182RYC2**	62324.A0A182RYC2	Serine protease inhibitor (serpin) 9; Belongs to the serpin family.
**A0A182RYH7**	62324.A0A182RYH7	Gustatory receptor; Gustatory receptor which mediates acceptance or avoidance behavior, depending on its substrates; Belongs to the insect chemoreceptor superfamily. Gustatory receptor (GR) family.
**A0A182RZI4**	62324.A0A182RZI4	Calreticulin
**A0A182RZL2**	62324.A0A182RZL2	28S ribosomal protein S26, mitochondrial
**A0A182S534**	62324.A0A182S534	Cytochrome P450; Belongs to the cytochrome P450 family.
**A0A182S535**	62324.A0A182S535	Cytochrome P450; Belongs to the cytochrome P450 family.
**A0A1I8JUP1**	62324.A0A1I8JUP1	Odorant-binding protein 8
**A0A1Y9HDW6**	62324.A0A1Y9HDW6	Heme peroxidase 1

Table 6: The table displays the corresponding *An. funestus* vector IDs (AFUN_id) and String IDs (uniprot_id), along with their significant fold change (FC) values.

vector_id	uniprot_id	FC
AFUN000380	A0A182R2I9	3,65
AFUN001070	A0A182R4H8	2,41
AFUN001382	A0A182R4H8	2,87
AFUN002194	A0A182R7P4	-2,14
AFUN002275	A0A182R7X5	-2,41
AFUN002308	Q06DF8	2,04
AFUN002414	A0A182R8B2	-3,01
AFUN002978	A0A4Y0AR66	2,51
AFUN003140	A0A182RAC6	-2,87
AFUN003738	A0A182RC23	-2,62
AFUN003850	A0A182RCD4	3,39
AFUN003917	A0A182RCJ8	5,33
AFUN004479	A0A182RE57	-2,39
AFUN004884	A0A182RFA6	-2,97
AFUN005101	A0A182RFW9	-5,36
AFUN005102	A0A182RFX0	-2,54
AFUN005103	A0A182RFX1	-3,48
AFUN005196	A0A182RG59	-2,01
AFUN005751	A0A182RHN9	3,06
AFUN006135	A0A182RIS0	2,78
AFUN006610	A0A182RK42	2,06
AFUN006671	A0A182RKA3	5,02
AFUN006702	A0A182RKD4	2,44
AFUN006859	A0A182RKU1	-5,72
AFUN006915	A0A182RKZ7	3,24
AFUN007013	A0A182RL95	-4,8
AFUN007549	A0A182RMS7	6,97
AFUN007717	A0A182RN86	-4,18
AFUN007741	A0A182RNB0	-2,43
AFUN008275	A0A182RPU2	-2,27
AFUN008299	0A182RPW6	-2,53
AFUN008426	A0A182RQ85	-3,21
AFUN008829	A0A182RRD4	-3,78
AFUN010604	A0A182RWE4	-2,29
AFUN010903	A0A182RX91	-2,16
AFUN010918	A0A182RXA4	3
AFUN011055	A0A182RXN7	-4,94
AFUN011064	A0A182RXP6	-7,02
AFUN011238	A0A182RY65	-2,2
AFUN011295	A0A182RYC2	-2,79
AFUN011350	A0A182RYH7	-7,12
AFUN011715	A0A182RZI4	-2,25
AFUN015739	A0A182S4L5	2,24
AFUN015785	B5AIH8	3,01
AFUN015786	B5AIH9	3,8
AFUN015790	A0A182S4R9	2,13
AFUN015792	B5AII6	3,53
AFUN015801	5AII2	2,57
AFUN015802	B5AII1	2,84
AFUN015810	A0A182S4T9	2,21
AFUN015853	A0A182S4Y3	3,02
AFUN015854	0A182S4Y4	4,17
AFUN015889	B5AII7	5,28
AFUN015894	0A182S524	-5,01
AFUN015904	A0A182S534	-2
AFUN015905	A0A182S535	-3,39
AFUN015919	A0A182S550	2,09
AFUN016174	A0A1I8JUP1	-20,53
AFUN016374	A0A1Y9HDQ3	4,14
AFUN016429	A0A1Y9HDU8	2,43
AFUN016430	A0A1Y9HDV4	2,14
AFUN016444	A0A1Y9HDW6	-7,04
AFUN017067	A0A182R6B0	2,1
AFUN017471	A0A182RW11	2,16
AFUN017538	A0A182RW78	2,32
AFUN017592	A0A182RMX0	2,23
AFUN017593	A0A182RMX1	2,06
AFUN017611	A0A182RWF1	2,32
AFUN017743	A0A182RZL2	-3,98
AFUN017757	A0A182S4N6	3,31
AFUN017914	A0A182S031	3,35

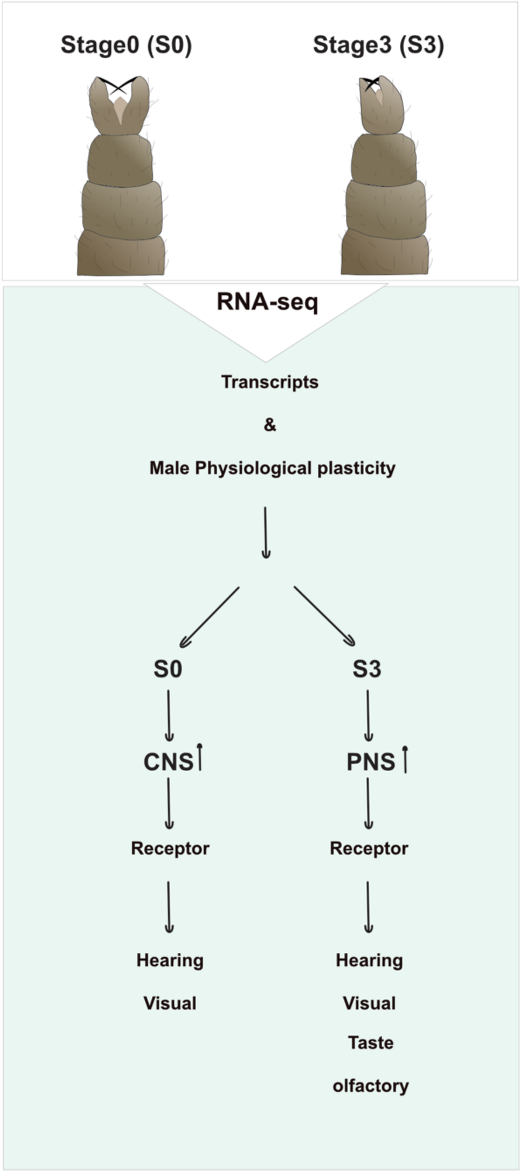

Supplementary Fig 3. Schematic model of male mosquito gene expression during the sexual development period immature stage (S0 = no rotation of the genitalia) and maturing stage (S3 = 90° - 135° rotation of the genitalia) affects the male mating ability and mosquito population stability, which will directly improve the odds of pathogen transmission. During male genitalia rotation and sexual maturity, the regulation of nervous system process was highly maintained. However, the proportion of differentially abundant transcripts for central nervous system process (CNS), and sensory perception of sounds and vision increased in immature stage (S0), while there was a significant announced shift towards peripheral nervous system process (PNS) in maturing stage (S3) such as the olfactory receptor activity, signalling receptor activity, odorant binding, photoreceptor activity, taste receptor activity, and small molecule sensor activity.
